# Clinical implications of *alpha*, *beta*, and *gamm*a HPV infection in juvenile onset recurrent respiratory papillomatosis

**DOI:** 10.1007/s00405-021-07040-9

**Published:** 2021-08-28

**Authors:** Martina Bertinazzi, Tarik Gheit, Jerry Polesel, Sandrine McKay-Chopin, Cesare Cutrone, Marianna Sari, Marta Sbaraglia, Angelo Paolo Dei Tos, Piero Nicolai, Massimo Tommasino, Paolo Boscolo-Rizzo

**Affiliations:** 1 Department of Neurosciences, Section of Otolaryngology, University of Padova, Padova, Italy; 2 International Agency for Research On Cancer, Lyon, France; 3 Unit of Cancer Epidemiology, Centro Di Riferimento Oncologico Di Aviano (CRO) IRCCS, Aviano, Italy; 4 Department of Medicine, Section of Pathology, University of Padova, Padova, Italy; 5 Department of Medical, Surgical and Health Sciences, Section of Otolaryngology, University of Trieste, Strada di Fiume 447, 34149 Trieste, Italy

**Keywords:** Juvenile respiratory papillomatosis, Human papillomavirus, Co-infections, Children, Prognosis

## Abstract

**Purpose:**

The aim of our study was to evaluate the prevalence of different HPV genera—*alpha, beta* and *gamma*—in Juvenile onset Recurrent Respiratory Papillomatosis (JoRRP) and examine the association of type and genus-specific viral features with the clinical outcome of disease.

**Methods:**

This retrospective observational study included consecutive patients with JoRRP who were treated in a referral centre between October 2000 and October 2020. All patients underwent cold excision and laser vaporisation of papillomatous lesions. Samples were analysed for the presence of 120 viral genotypes (22 alpha-HPV, 46 beta-HPV, 52 gamma-HPV) using a highly sensitive multiplex genotyping assay.

**Results:**

Twenty patients with JoRRP, aged 0.3–11 years, were included, with a median follow-up of 13.5 years. All samples were HPV DNA positive: 20 (100%) for alpha-HPV DNA; 7 (35%) for beta—HPV DNA; 0 for gamma-HPV DNA. Three groups were defined according to the number of infections: seven cases (35%) with HPV mono-infection; ten cases (50%) with HPV double-infection; three cases (15%) with ≥ 3 HPV infections. At diagnosis, patients with ≥ 3 HPV infections reported higher median Derkay’s score than those with mono-infection (21 vs 14, *P* = 0.018). Number of HPV infections was also associated with clinical outcomes, with an average of 0.5 surgical procedures/year in patients with mono-infection, 1.2 for double-infection, 2.6 for ≥ 3 infections (*P* = 0.006).

**Conclusion:**

Despite the small sample size, these preliminary data support an association between the number of different alpha and beta HPV co-infections and the clinical severity of the disease.

## Introduction

Juvenile onset recurrent respiratory papillomatosis (JoRRP) is a rare and challenging HPV-induced disease, frequently associated with mucosal HPV-type infection, in particular HPV 6 and 11, and characterised by the growth of squamous papillomata in the airway epithelium [1]. It is defined by the onset before 12 years of age with the peak of incidence being commonly before 5 years [2]. Because of JoRRP’s typical recurrence, patients often require multiple surgical debulking procedures to maintain an airway patency [3].

There is currently no eradicative cure for JoRRP. Furthermore, no reliable markers predicting the course of the disease, the risk of recurrence or malignant transformation exist [4]. However, the new adjuvant therapies appear to be promising in improving the control of the disease [5]. It could be, therefore, interesting and innovating having biological markers that can guide clinicians to select patients eligible for adjuvant therapies.

Many studies have investigated whether the severity and aggressivity of JoRRP may be influenced by the infection with mucosal HPV types, 6 and 11, but the results provided inconsistent and contrasting conclusions [1, 6]. To the best of our knowledge, no study has ever investigated the prevalence of HPV genera other than mucosal alpha genus in JoRRP. Interestingly, some beta HPV types from species 3 appears to have dual tropism, being detected in nasal or anal mucosa and the skin [7, 8]. Thus, in this study we have evaluated the prevalence of different HPV genera—alpha, beta and gamma, in JoRRP with a highly sensitive genotyping assays and investigated whether different pattern of HPV infection may influence the clinical outcome of the disease.

## Materials and methods

### Study population

This observational study included consecutive children aged < 12 years at onset of JoRRP, in a referral centre (Section of Otolaryngology, University of Padova) between October 2000 and October 2018 and followed-up until October 2020. A clinical and surgical database of all patients, including age and Derkay’s score [9] at onset, yearly number of surgical procedures since the diagnosis, duration of follow-up, surgical and controls videos, surgical and clinical outcomes have been maintained prospectively since October 2000. All patients were treated with both cold surgical and laser vaporisation with active aspiration by the same expert surgeon (CC) in the same single centre. The whole study was approved by the ethic committee for clinical experimentation of Treviso and Belluno provinces (Ethic votes: 345/AULSS9 and 421/AULSS9).

Inclusion criteria comprehended: (a) diagnosis of JoRRP; (b) age ≤ 12 years old at onset; (c) surgical treatment; (d) histological and clinical data available in hospital database; (e) written consent for data processing.

From October 2000 to October 2018, 23 children age ≤ 12 years were diagnosed with JoRRP and underwent surgical treatment. Three cases were excluded due to lack of tissue specimen for analysis. Thus, the final case series included 20 patients. Depending on clinical course of the disease, considering the number of surgical procedures/year and the presence of a longitudinal progression of papillomatosis, we stratified the patients in three groups: (a) *in remission*; (b) *with persistence*; (c) *in progression*. We defined *in remission* patients with no evidence of papillomata in the past 5 years; *with persistence* the patients submitted to less than 4 surgical procedures in the last year and/or without a longitudinal progression of papillomatosis, and *in progression* the patient submitted to more than or equal to 4 surgical procedures in the last year and/or with a longitudinal progression of papillomatosis.

### Collection of samples and DNA extraction

Paraffined tissue samples for molecular analysis were obtained from surgical specimens. Micro slices of all paraffined samples were shipped to the Infection and Cancer Biology Group at the International Agency for Research on Cancer in Lyon, France. DNA was obtained by an overnight incubation at 37 °C of the paraffin tissue sections in a digestion buffer (10 mM Tris/HCl pH 7.4, 0.5 mg/ml proteinase K, and 0.4% Tween 20) [10]. To monitor the possible occurrence of cross-contamination between the different specimens during DNA extraction, tubes containing only buffer were also included (one tube with buffer every 10 specimens).

### HPV type-specific PCR bead-based multiplex genotyping assay (TS-MPG)

The identification of 120 viral genotypes was performed by using a high sensible type—specific multiplex genotyping assays (TS-MPG), which combine multiplex polymerase chain reaction (PCR) and bead-based Luminex technology (Luminex Corp., Austin TX, USA) as described in the literature [11, 12]. Multiplex type-specific PCR uses specific primers for the detection of 22 alpha-papillomavirus types (HPV6, 11, 16, 18, 26, 31, 33, 35, 39, 45, 51, 52, 53, 56, 58, 59, 66, 68^a and b^, 70, 73 and 82), 46 beta-papillomavirus types (HPV5, 8, 9, 12, 14, 15, 17, 19, 20, 21, 22, 23, 24, 25, 36, 37, 38, 47, 49, 75, 76, 80, 92, 93, 96, 98, 99, 100, 104, 105, 107, 110, 111, 113, 115, 118, 120, 122, 124, 143, 145, 150, 151, 152, 159, 174) and 52 gamma–papillomavirus types (HPV4, 48, 50, 60, 65, 88, 95, 101, 103, 108, 109, 112, 116, 119, 121, 123, 126, 127, 128, 129, 130, 131, 132, 133, 134, 148, 149, 156, 161, 162, 163, 164, 165, 166, 167, 168, 169, 170, 171, 172, 173, 175, 178, 179, 180, 184, 197, 199, 200, 201, 202). Primers for the amplification of beta-globin were also added to provide a positive control for the quality of the template DNA. Amplification of beta-globin gene showed that in all cases the samples contained good quality DNA. The results were expressed as mean fluorescence intensity (MFI) [13]. The cut-off was computed by adding 5 MFI to 1.1 times the median background value.

### Statistical analysis

Differences in socio-demographic and clinical characteristics of the patients across strata were assessed with the Kruskal–Wallis test or Fisher’s exact test. The values of *P* reported in the tables were considered significant when ≤ 0.05. For each patient and for each year after diagnosis, the cumulative number of surgical procedures was calculated as the total number of surgical procedures from the diagnosis.

## Results

### Clinical results

Patients’ clinical data were included in Table 1. The median age at diagnosis was 3.0-years old (0.3–11.0-years old) and the male:female ratio was approximately 1:2. The median duration of follow-up was 13.5 years (2–20 years) and the Derkay’s score at onset (clinical and anatomical score) varied between 5 and 23 with a median value of 14. Papillomata demonstrated different localizations along the airways with different prevalence. As shown in Table 2*,* the larynx was the most involved site (95% of cases). The median number of procedures/year at onset was 2, ranging between 1 and 10. The total number of surgeries ranged from 1 to 45 with a median value of 9.5, while the maximum number of surgeries/year ranged from 1 and 13, with a median value of 3. Three patients received adjuvant cidofovir and two patients received indole-3 carbinol following surgical treatment for recurrence JoRRP.

**Table 1:** Patients’ clinical, socio-demographic and viral characteristics

#	Sex	Age at onset (years)	Derkay’s score at onset	Follow-up (y)	Number of HPV co-infections	Alpha-HPV	Beta-HPV
10	F	2.8	14	12	1	HPV6	–
17	F	2	10	4	1	HPV6	–
18	F	3	16	14	1	HPV6	–
19	F	11	7	2	1	HPV6	–
12	M	2	9	7	1	HPV11	–
9	F	5.3	5	5	1	HPV11	–
16	F	3	9	19	1	HPV11	–
15	F	0.8	16	15	2	HPV6	HPV17
3	F	0.3	17	10	2	HPV6	HPV19
4	M	1.1	11	14	2	HPV6	HPV111
7	F	4	9	19	2	HPV6	HPV111
1	M	3.2	6	20	2	HPV6, HPV11	–
13	M	6	20	19	2	HPV6, HPV11	–
14	F	1.5	19	14	2	HPV6, HPV11	–
2	M	4	15	10	2	HPV11, HPV16	–
8	F	2.1	14	9	2	HPV11, HPV16	–
11	F	3	7	13	2	HPV11, HPV16	–
5	M	4	23	15	3	HPV6, HPV11	HPV111
20	F	3	19	6	3	HPV6, HPV11	HPV100
6	M	2	21	19	4	HPV11, HPV16	HPV21, HPV100

**Table 2:** Clinical and socio-demographic characteristics at diagnosis according to number of co-infections

	All		HPV co-infection						*p* value^a^
			One		Two		Three or more		
	*n*	(%)	*n*	(%)	*n*	(%)	*n*	(%)	
Total	20	(100)	7	(35)	10	(50)	3	(15)	
Age at diagnosis (years)									
Median	3.0		3.0		2.6		3.0		0.720
Min, max	(0.3, 11.0)		(2.0, 11.0)		(0.3, 6.0)		(2.0, 4.0)		
Sex, female	13	(65)	6	(86)	6	(60)	1	(33)	0.216
Localization									
Pharynx	2	(10)	1	(14)	1	(10)	0	(0)	1.000
Larynx	19	(95)	6	(86)	10	(100)	3	(100)	0.500
Trachea	5	(25)	1	(14)	2	(20)	2	(67)	0.228
Bronchus	1	(5)	0	(0)	0	(0)	1	(33)	0.150
Lung	1	(5)	0	(0)	0	(0)	1	(33)	0.150
Multiple	6	(30)	1	(14)	3	(30)	2	(67)	0.274
Derkay’s score at diagnosis									
Median	14		9.0		14.5		21		0.018
Min, max	(5, 23)		(5, 16)		(6, 20)		(19, 23)		
Duration of follow-up (year)									
Median	13.5		7.0		14.0		15.0		0.159
Min, Max	(2, 20)		(2, 19)		(9, 20)		(6, 19)		

^a^Estimated according to Kruskal–Wallis test of Fisher’s exact text, as appropriate

All samples were HPV-DNA positive and they reported at least one infection for alpha-HPV DNA; seven cases (35%) were also positive for beta-HPV DNA, while no gamma-HPV DNA was detected in any specimen (Table 1).

### Results of bio-molecular investigations

Depending on the number of infections, patients were categorised into three groups (Table 2): (1) patients with HPV mono-infection (seven cases, 35%); (2) patients with HPV double-infection (10 cases, 50%); (3) patients with ≥ 3 HPV infections (three cases, 15%). Thus, 65% of patients have multiple HPV infections. Of the 10 samples with double co-infection, 6 (30%) showed an alpha-HPV co-infection (three patients with HPV6-11 and three patients with HPV11-16) while four samples (20%) showed alpha and beta co-infection (HPV6-19; HPV6-111; HPV6-111; HPV6-17). Finally, the three samples (15%) with co-infection of ≥ 3 papillomavirus genotypes were all positive for alpha HPV11 associated with another alpha genotype (HPV6 or HPV16) and with an HPV beta genotype ranging in number from 1 to 2 (HPV6-11-100; HPV6-11-111; HPV11-16-21-100). The maximum number of co-infections observed in the present series was 4.

### Clinical and virological correlations

The three groups showed similar age at onset. In all groups, the main site of papillomata was the larynx, involved in 100% of cases with co-infection and in 86% of patients with mono-infection. Although not significant, multiple localization was more frequent in patients with ≥ 3 HPV infections than those with one or two co-infections (67% vs 30%, respectively). The Derkay’s score at diagnosis increased with the increasing number of co-infections, reaching 21 for ≥ 3 HPV co-infection compared to 9 for patients with mono-infection (*P* = 0.018) (Table 2). The median number of surgical interventions at onset was higher for patients with ≥ 3 multiple infections, though not significant (Table 3). However, the maximum number of surgical procedures/year (*P* = 0.012), the average number of surgical procedures/year (*P* = 0.022) and the total number of surgeries (*P* = 0.006) increased with increasing number of HPV co-infections. Three patients were considered *in progression* and they were all co-infected patients: one positive for double co-infection and two positives for multiple co-infections. The majority of patient with HPV mono-infection (57%) was clinically *in remission*, whereas, no one showed progression (Table 3).

**Table 3:** Clinical history according to number of co-infections

	All		HPV co-infection						*p* value^a^
			One		Two		Three or more		
	*n*	(%)	*n*	(%)	*n*	(%)	*n*	(%)	
Total	20	(100)	7	(35)	10	(50)	3	(15)	
Number of surgeries/year at diagnosis									
Median	2.0		2.0		2.0		8.0		0.387
Min, max	(1, 10)		(1, 3)		(1, 7)		(1, 10)		
Maximum number of surgeries/year									
Median	3		2.0		3.5		8.0		0.012
Min, max	(1, 13)		(1, 3)		(1, 13)		(4, 10)		
Average number of surgeries/year									
Median	1.2		0.5		1.2		2.6		0.022
Min, max	(0.1, 4.3)		(0.1, 2.3)		(0.2, 1.7)		(2.4, 4.3)		
Total number of surgeries									
Median	9.5		3.0		15		39		0.006
Min, max	(1, 45)		(1, 10)		(2, 28)		(26, 45)		
Clinical outcome									
Remission	10	(50)	4	(57)	6	(60)	0	(0)	0.161
Persistence	7	(35)	3	(43)	3	(30)	1	(33)	
Progression	3	(15)	0	(0)	1	(10)	2	(67)	

^a^Estimated according to Kruskal–Wallis test of Fisher’s exact text, as appropriate

**Fig. 1:**
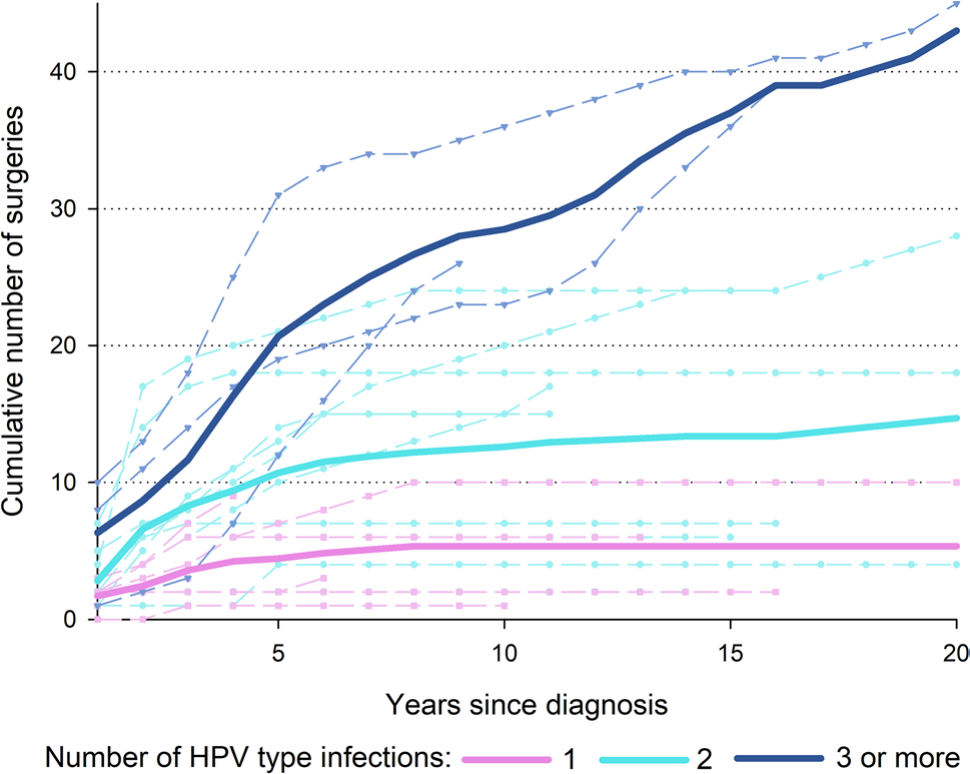
Cumulative number of surgeries according to number of co-infection with *alpha*-HPV and with *beta*-HPV

**Fig. 2:**
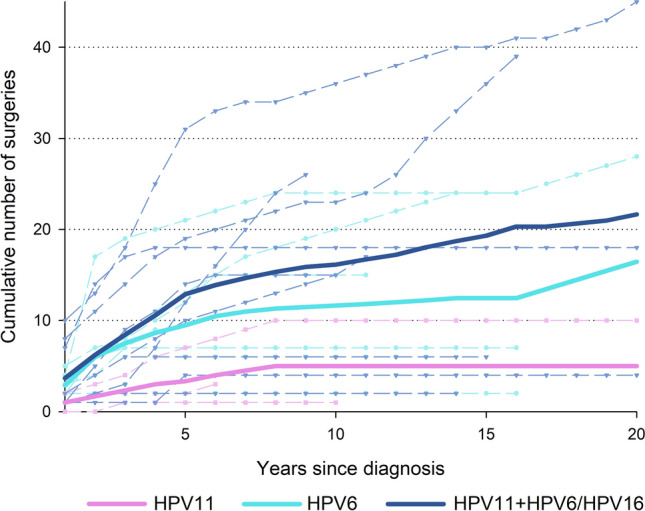
Cumulative number of surgeries according to *alpha*-HPV infections

Clinical outcome was also evaluated as required number of surgical procedures after diagnosis. Interestingly, the average cumulative number of surgical procedures flattened after approximately 5 years from onset for patients with mono-infection or double-infections, even if at a lower level for the former than the latter (average cumulative number = 5.3 and 14.7, respectively). Conversely, patients with ≥ 3 HPV infections showed an upward trend over the all period of observation, reaching an average cumulative number of 43.0 surgical procedures after 20 years from diagnosis (Fig. 1). Cumulative number of surgical procedures was also calculated using information on HPV6 and HPV11 alone (Fig. 2). Patient with HPV6/HPV11 co-infection showed the worse clinical course, whereas those with HPV11 infection showed the best clinical course.

## Discussion

To the best of our knowledge, this is the first study that analysed the presence of a large number of alpha, beta and gamma HPVs types in JoRRP using a highly sensitive method. The data show that all JoRRP were associated with infections of mucosal alpha HPV types 6 and 11, which are well-known etiological factors of JoRRP. In addition, several JoRRP were also positive for some beta HPV types. Conversely, no gamma HPV types were detected in this series. Although this may be related to the limited number of cases analysed, the prevalence of gamma HPV infection of the female genital tract and gamma HPV transmissibility is much lower than that of alpha and beta HPV [14].

Despite the small study size, the findings highlight an association of multiple HPV infections with the severity of disease in comparison to single HPV infections.

The main problems of recurrent respiratory papillomatosis are still the lack of an eradicating treatment, its unpredictable nature, and the limited knowledge on the pathogen-host interaction [15]. This lack of information has prompted many clinicians to try to provide a prognosis of the disease, looking for the risk factors most involved in the aggressiveness of RRP. Most of the available studies in literature found a correlation between the aggressiveness of RRP and the aetiology by HPV11 [16].

It has been assumed that HPV11 E7 may interact more effectively with the transporter associated with antigen processing subunit 1 (TAP1) than HPV6 E7, leading to reduced immune responses and the more severe disease course observed in HPV11 RRPs [17, 18]. However, other studies did not provide evidence for the higher aggressive properties of HPV11 versus HPV6^1^. Indeed, some studies reached opposite conclusions, being HPV6 more associate with higher JoRRP severity in comparison to HPV11 [19, 20]. Other studies highlight that age at diagnosis, rather than the genotype of HPV involved, can impact on the clinical history of the disease [1, 6].

Our findings showed that single infections have a lower prevalence than co-infections and that the latter have a much more aggressive outcome than the others which instead tend to stabilise after a follow-up of about 4 years (Fig. 1). Considering the results of prevalence of HPV genera (alpha–beta–gamma) and the clinical data, it is evident that in the present case series an increased number of co-infections was associated with increasing aggressiveness of the disease determined both by the number of surgical procedures and the longitudinal spread of the disease (Fig. 1)*.* On the other hand, by considering the clinical course of papillomatosis with respect to alpha-HPV infection only, it was observed that HPV11 is the genotype involved in a more aggressive course of the disease. However, it was clear only when HPV11 was associated with other viral genotypes (co-infections) but not in cases of mono-infection, where instead the disease has a more indolent course (Fig. 2).

Multiple studies concerning the biological mechanism and the role of interactions and co-infections between different HPV genotypes were conducted on other widely spread HPV-related tumours [21]. Primarily on cervical cancer, about which several authors agree on the association between genotype-specific HPV interactions, multiple viral co-infections increase risk of malignant trasformation. [22] Concerning respiratory papillomatosis, in 2010 Donne and colleagues did not find a certain clinical difference between HPV6 and HPV11 infections and recognised the possible impact of untested HPV-variants co-infections on the clinical outcome of RRP [23].

Other authors had also highlighted the presence of co-infections in respiratory papillomatosis, considering both other viruses—such as EBV, HSV and CMV, and other alpha-HPV genotypes, assuming their co-presence as an additional risk factor for the clinical outcome of the RRP [24]. In 2015 Omland and colleagues decided to stratify RRP patients depending on alpha-HPV viral load without considering HPV co-infections. They finally demonstrate the absence of a correlation between the RRP prognosis and the HPV viral load, defined as irrelevant if < 5 copies/cell [6]. Other authors, such as Buchinsky, excluded cases of alpha-HPV-DNA co-infections from their cluster because, in their opinion, it was impossible to determine which of the different involved genotypes was responsible for the clinical phenotype of the disease [1].

However, the data of the present study do not provide any evidence of a direct role of co-infections in determining the greater aggressiveness of the disease. It has been observed that immunosuppressed patients often harbour multiple oral HPV infections [25]. Thus, the association between the aggressiveness of JoRRP and multiple HPV infections may reflect patient’s immuno-suppressive status which more directly may affect the aggressive course of the disease.

Limited sample size is the major limitation of the present study, and, therefore, the results should be considered as preliminary. JoRRP is an extremely rare disease. Considering that our centre serves a population of 775,000 children under the age of 12, the estimated incidence of 0.15 per 100 000 child/year is in line with the data reported in the literature [26]. Furthermore, we cannot evaluate the impact of adjuvant treatment on multiple recurrences rate due to the low number of patients receiving additional medical treatment after surgical excision. Nonetheless, results are strongly indicative of a new prognostic trend in JoRRP that would benefit the inclusion of a larger sample for further validation. Indeed, small sample size mainly impact on false negative results as the study is not powered to detect small differences. Further, the small sample size did not allow multivariable analyses. Conversely, the homogeneity of patients, treated and followed up by the same centre, is to be account among study strength.

## Conclusions

JoRRP still remains an investigating pathology in terms of treatment and clinical course. Both alpha and beta HPV genera may be involved in JoRRP pathogenesis and the number of different HPV-type co-infections may serve as a prognostic indicator of the clinical course of the disease. The results of this study, although preliminary and based on a small number of patients, underline the importance of a highly sensitive diagnostic method in this pathology. However, further studies are needed to confirm our observations.

## Declarations

### Availability of data and material

All data and materials used in this study are now available in clinical database.
